# Biological control of human disease vectors: a perspective on challenges and opportunities

**DOI:** 10.1007/s10526-017-9815-y

**Published:** 2017-05-10

**Authors:** Matthew B. Thomas

**Affiliations:** Department of Entomology and Center for Infectious Disease Dynamics, Penn State, University Park, PA 16802 USA

**Keywords:** Vector-borne diseases, Insecticide resistance, Biocontrol, Mosquito, Integrated Vector Management

## Abstract

Chemical insecticides are the mainstay of contemporary control of human disease vectors. However, the spread of insecticide resistance and the emergence of new disease threats are creating an urgent need for alternative tools. This perspective paper explores whether biological control might be able to make a greater contribution to vector control in the future, and highlights some of the challenges in taking a technology from initial concept through to operational use. The aim is to stimulate a dialogue within biocontrol and vector control communities, in order to make sure that biological control tools can realize their full potential.

## Introduction

Contemporary control of human disease vectors relies almost exclusively on insecticide-based interventions. This situation is especially true for malaria vectors, but applies also to vectors of other human diseases such as dengue, Zika, Chagas, leishmaniasis, etc., as well as to some livestock diseases. This paper examines whether biological control could play an increased role in vector control and contribute to the development of sustainable Integrated Vector Management (IVM) strategies in which diverse tools, tactics, and resources are combined to reduce transmission of disease by vectors. Looking at parallels with agriculture and the established role of biological control in development of Integrated Pest Management (IPM) strategies, a role in IVM ought to be possible (Thomas et al. 2012). Yet in reality there are very few examples where biological control is playing an established role in vector control. This paper examines why this might be the case. The approach is not to present a review of the diverse research studies conducted over the years on biological control approaches, in part because this literature is reviewed elsewhere (e.g., Chapman 1985; McGraw and O’Neill 2013; Benelli et al. 2016; Huang et al. 2017; Saldaña et al. 2017). Rather, this paper aims to highlight some of the opportunities but also the challenges in developing a biological control tool or tactic to the point of operational use.

The paper is framed around the following assumptions and caveats:

(i) The insights derive largely from work on the ecology and control of adult malaria mosquitoes. Other disease vectors, such as the *Aede*s spp. responsible for transmitting dengue, chikungunya and Zika viruses, have different ecologies and so control tools or approaches might not necessarily transfer between systems. However, the key challenges for developing a control tool through to operational use (namely demonstrating efficacy, defining a clear role within IVM, regulatory approval and implementation) are similar across vector borne disease systems.
(ii) The definition of biological control used in this paper centers on the use of predators, parasites, or pathogens, where the mode of action relies on the living organisms and not simply their derivatives. This definition puts pesticides based on the toxin-forming bacteria *Bacillus thuringiensis* and *B. sphaericus* at the margins of biological control, which is not to say they are not valuable technologies, but the aim of this paper is to look at opportunities beyond these established products. Use of toxins derived from plants or microbes, or insecticide juvenile hormone analogues are not considered biological control, even though these are often referred to as ‘biological’ products.
(iii) The definition of biological control also excludes transgenic and gene drive technologies where the vector itself is genetically modified for the purpose of population suppression or population replacement strategies, since there is no natural enemy involved. Conventional sterile insect technique is excluded for similar reasons. Other recent reviews (e.g., Benelli et al. 2016; Huang et al. 2017) have taken a more pluralistic view of biological control to include these ‘biologically-based’ strategies. However, the long-standing definition of biological control centers on the use of a natural enemy and not modification of the pest itself. This definition is consistent with the policy of the current journal and the International Organisation for Biological Control (IOBC). On the other hand, novel approaches like trans-infection of vectors with an endosymbiont such as *Wolbachia* (McMeniman et al. 2009; Hoffmann et al. 2011), or paratransgenisis, where a pathogen or parasite is genetically modified in some way to affect the capacity of the vector to transmit the target disease (Wilke and Marrelli 2015), are included as they do use a living parasite to effect change in another target organism.

## Opportunities for new tools for the control of disease vectors

Recent years have seen dramatic reductions in the burden of malaria worldwide. The decline is largely attributable to the broad-scale use of long lasting insecticide treated nets (LLINs) and indoor residual sprays (IRS) against adult mosquitoes (Bhatt et al. 2015). The success of these insecticide-based interventions represents a foundational step in the global agenda to eliminate malaria. However, the current control tools alone are likely insufficient to eliminate malaria in many settings, even if their use could be intensified further (malERA Consultative Group on Vector Control 2011; WHO 2015; Griffin et al. 2016). Additionally, there is a growing problem of insecticide resistance that could render existing tools ineffective and potentially lead to a reversal of recent gains (WHO 2015; Griffin et al. 2016; Hemingway et al. 2016).

Similar challenges exist for control of arboviruses such as dengue, chikungunya and Zika, which have emerged as major threats to public health in recent years. In the absence of effective drugs and vaccines, the ability to combat these diseases relies on vector control (Yakob and Walker 2016). Again, chemical insecticides, the current mainstay, are being undermined by the evolution of insecticide resistance (Lima et al. 2011; Bellinato et al. 2016; Duong et al. 2016; Ishak et al. 2017). Moreover, the key mosquito vectors of these arboviruses, *Aedes aegypti* and *A. albopictus*, continue to spread into new areas, including parts of Europe and the US (Kraemer et al. 2015). Other emerging diseases, such as West Nile virus (transmitted by a diversity of mosquito species) (Paz 2015) and Lyme disease (transmitted by certain ticks) (Ostfeld and Brunner 2015) are also extending the threat of vector-borne diseases into temperate environments not typically associated with vector borne disease problems in recent history. These challenges create a demand for new control tools, and could increase opportunities for biological control.

## Building a role for biological control

### Making the case

There is no reason a priori why biological control technologies cannot offer new tools and strategies, and contribute to the development of IVM strategies that reduce the reliance on insecticide-based interventions. However, the track record of biological control in vector control is limited, especially if we exclude the bacterial pesticides *B. thuringiensis* and *B. sphaericus*. Without a pedigree, there is no major incentive for the public health community to look to biological control for answers. Many decades of chemical control have created a pesticide mindset. Similar mindset limits the adoption of biological control tools in certain agricultural sectors (van Lenteren 2012).

The ‘organic’ or ‘green’ economic premiums that can help drive the adoption of biological control approaches in agriculture and natural environments do not carry the same weight in vector control. National vector borne disease control programs tend to operate on limited budgets within the context of public health systems. In many settings, the householders make no direct financial contribution for an intervention so there is minimal capacity to pass on control costs to the ‘consumer’. Furthermore, while an individual householder can make a choice to buy organic produce, that same householder cannot choose the nature of the vector control products or strategy delivered at the community level. Perhaps most fundamentally, current vector control is dominated by short-term economics, with products most frequently purchased via a tender process that emphasizes minimum cost for maximum coverage. This approach constrains innovation and provides little incentive for technologies that might have added value or contribute to a ‘public good’, such as increased environmental or evolutionary sustainability.

Additionally, there are many ways to kill a mosquito or otherwise disrupt transmission. Developing novel chemical actives is an obvious route and has been a primary focus of initiatives such as the Innovative Vector Control Consortium (IVCC) (Hemingway et al. 2006). Diverse alternative technologies such as toxic sugar baits (Müller et al. 2010), house screening/modification (Kirby et al. 2009; Knols et al. 2016), endectocides (Chaccour et al. 2013), repellants (Achee et al. 2012), lethal ovitraps (Paz-Soldan et al. 2016), mass trapping (Homan et al. 2016), genetic control strategies (reviewed in McGraw and O’Neill 2013), etc. are all the subject of ongoing research. Against this backdrop, it is not obvious why either industry or government should single out biological control solutions over other alternative technologies. Research and Development (R&D) budgets are finite and there are likely trade-offs in funding at some level. The fact that a biological control technology ‘can’ be developed is not necessarily sufficient. There needs to be a justification for why a biological control technology ‘should’ be developed. Depending on the particular approach and system, the reasons could be manifold, including: self–sustaining, self–spreading, non-chemical, lack of any alternative tool, resilient to evolution of resistance, overcoming insecticide resistance, cheap, novel delivery systems, complementary to conventional tools, targeting residual transmission, potential for community engagement, local production and ownership, donor-driven, etc. Whatever the reason(s), the case needs to be clear.

### Demonstrating efficacy

A frequent starting point for developing a technology is to evaluate it in the laboratory. If it doesn’t work in the laboratory, where conditions are highly controlled, it probably isn’t going to work in the field. However, a substantial and rigorous body of evidence is required to obtain approval and ultimate use of a technology or approach by the likes of The World Health Organisation (WHO), funding agencies, regulatory authorities and national programs.

The generally accepted gold standard for demonstrating efficacy of a public health intervention is a randomized controlled trial (RCT) with epidemiological end points. Building a product or approach and progressing from basic phase I lab studies, through phase II semi-field studies, up to a full-blown phase III RCT is challenging. It takes time, multiple granting successes and substantial resources. This situation is not unique to biological control and is recognized as a major challenge for development of novel public health tools. For new tools that fit with established paradigms, there are efforts to streamline the evaluation pathway (Vontas et al. 2014). For example, a new chemical active ingredient for use in indoor residual spraying (IRS) might not need a phase III trial as IRS itself is a proven approach and whether the new active performs better than an existing product can likely be demonstrated in a phase II equivalency test. However, where the paradigms are novel and there are no clear reference points, evidence of epidemiological impact is likely required (Vontas et al. 2014).

While the need for rigorous evaluation is also true in agriculture, there are some important differences between agriculture and public health. First, although not universally true, demonstrating the impact of a pest control tool in agriculture might well be possible in relatively small replicated plots within or across individual fields or glasshouses. The epidemiology of diseases such as malaria or dengue tends to play out over larger scales. Second, agriculture is more accepting of intermediate end points, such as reductions in pest density, without necessarily demonstrating definitive impact on crop yield or profit margin for the grower. In public health, the primary end points usually relate to one or more measures of disease burden within the human population, and entomological end points alone are usual viewed as insufficient.

Very few biological control interventions for vector borne diseases have progressed to the level of evaluating epidemiological outcomes. Even the Eliminate Dengue Program (http://www.eliminatedengue.com/program), which is one of the largest and most high profile programs to explore a new mosquito control intervention in recent years, has yet to demonstrate epidemiological impact, though this research is ongoing. Moreover, many field studies examining intermediate entomological end points are poorly designed with insufficient power, inappropriate controls and inadequate monitoring. For example, biological control of mosquito larvae using fish has been researched for decades. In 2013 a Cochrane review was conducted to examine whether there was evidence that introducing larvivorous fish to anopheline breeding sites impacted malaria transmission or influenced adult anopheline density (Walshe et al. 2013). The review examined nearly 1300 published articles. There was not one reliable study that reported effects on malaria transmission. Only 12 studies were of sufficient quality to determine impacts on mosquito larval and pupal densities but the results themselves were inconclusive. The authors conclude that reliable research is insufficient to show whether introducing fish reduces transmission or density of malaria mosquito populations. Similarly, meta-analysis on use of invertebrate (copepod) larval predators against dengue vectors also reveals no clear evidence for policy recommendations (Lazaro et al. 2015).

Multiple proof-of-principle studies that provide evidence to support further development of a technology are not substitutes for proper efficacy trials. Fewer well-conducted trials (whether small or large-scale) would do more to strengthen the case for biological control than numerous poorly conducted trials. The fact that results might be mixed is not necessarily a bad thing, depending on the reasons for the variability. A technology that is inherently unreliable is not the same as a technology that works well but only does so under a certain set of ecological or socio-economic conditions (Klass et al. 2007). It is important that evaluation takes local context into account. Niche products could be very valuable as long as the niche can be characterized. There is now growing acceptance in the vector control community of the need for local optimization of technologies (Dicko et al. 2014; Mnzava et al. 2014; WHO 2015), a concept appreciated in biological control and IPM (e.g., Thomas et al. 2012; Harris et al. 2013; Parsa et al. 2014; Barzman et al. 2015; Guedes et al. 2016).

### Operational considerations

One component of technology development that is often neglected, particularly in initial stages of a project, is consideration of operational use. For example, numerous studies identify fungal pathogens as potential biological control agents for use against mosquito larvae (e.g., Pereira et al. 2009; Seye et al. 2013; Vogels et al. 2014; Greenfield et al. 2015; Alkhaibari et al. 2016). Yet there already exist non-chemical products, such as *B. thuringiensis* and *B. sphaericus*, which can work very well in certain settings (reviewed in Benelli et al. 2016). There are also numerous chemical insecticides, including insect growth regulators with modes of action distinct from standard neurotoxins (Devine et al. 2009; Devine 2016). However, the key challenge for larval control of mosquito vectors is not necessarily the lack of candidate products but the ability to deliver them in a cost-effective manner to breeding habitats that can be highly numerous, difficult to locate and transient. If the habitats cannot be treated because they are inaccessible or too numerous, or if the product requires frequent re-treatment because it does not persist, there is not obviously an advantage over current tools.

The need to consider operational use is not limited to larval control. For example, there is a growing interest in developing sugar baits as novel delivery systems for both chemical actives and biologicals (Müller et al. 2010; Beier et al. 2012; Marshall et al. 2013; Ondiaka et al. 2015; Dennison et al. 2016). One of the key challenges for this approach is whether artificial bait stations can compete against diverse sources of sugar available in the natural environment. Putting a biological control agent in a bait station does not necessarily address this problem. Furthermore, whether using a bait station to deliver something like a bacterium to alter susceptibility of mosquitoes to malaria parasites (Dennison et al. 2016) offers substantial advantages over a simple stomach poison which kills mosquitoes (males and females) directly is uncertain, not only in terms of efficacy but also in terms of key operational considerations such as production, formulation, supply chain and persistence.

These examples re-emphasize the need to identify what it is that a biological control approach is bringing to the table. Potential control benefits have to be weighed against operational challenges in order to define the overall rationale for development. Part of the rationale should involve a consideration of how much research and development is required to make the technology or strategy field-ready. Our capacity to continue to drive down malaria in the face of insecticide resistance requires new tools to be operational within the next 5–10 years (WHO 2015; Griffin et al. 2016). Given this pressing timeline, there is a strong argument for properly evaluating tools that have an existing R&D foundation on which to build. For example, in the last ten years there has been a substantial body of work exploring the potential for use of fungal pathogens against adult mosquitoes. The approach builds on the fact that commercial biopesticide products based on fungal pathogens exist already in agriculture. Numerous studies now provide evidence that fungal pathogens can reduce the vectorial capacity of mosquitoes (e.g. Blanford et al. 2005; Scholte et al. 2005; Lynch et al. 2012; Mnyone et al. 2012; Heinig et al. 2015); can infect diverse mosquito species, including strains resistant to insecticides (e.g. Scholte et al. 2007; Farenhorst et al. 2009; Howard et al. 2010; Blanford et al. 2011; Darbro et al. 2012); can be used in a variety of potential delivery strategies (e.g. Scholte et al. 2005; Blanford et al. 2012; Mnyone et al. 2012; Darbro et al. 2012; Carolino et al. 2014; Sternberg et al. 2016); and can satisfy important operational criteria such as storage, persistence and safety (e.g. Zimmermann 2007; Darbro and Thomas 2009; Blanford et al. 2012). There is little in this baseline research to suggest that products based on entomopathogenic fungi (which might be residual sprays or point source targets) could not be operationalized, and might be especially valuable in development of resistance management strategies requiring diverse products for use in rotations, mixtures or mosaics. Some additional effort towards large-scale field evaluation of fungi in the next 1–3 years, including operational feasibility and economic assessments, could provide the remaining evidence to either support implementation, or discount the approach once and for all. This is not to say that fungal pathogens are the only prospective tool with an existing research foundation. They are simply an illustration. Nor is it an argument that genuinely new approaches, which might take ten years or more to be field-ready, should not be explored. However, there is a tendency to be seduced by the next ‘big idea’, yet the timeline to implementation needs to be considered.

### Regulation and approvals

Funding for malaria control comes in large part from the donor community including The Global Fund, The World Bank, and bilateral country-to-country assistance. In order for products or interventions to receive donor support there is a general requirement that they have been evaluated to a sufficient standard to obtain recommendation from WHO. Initial evaluation of novel control tools that do not conform to conventional chemical insecticide target product profiles is the responsibility of the Vector Control Advisory Group (VCAG) (Vontas et al. 2014). The role of VCAG is to communicate with innovators on the development of early-stage vector control paradigms, assess the data to determine whether the evidence about the intervention is sufficient to justify its potential application, and make a recommendation to the Malaria Policy Advisory Committee of the Global Malaria Programme and/or the Strategic and Technical Advisory Group of the Department of Control of Neglected Tropical Diseases for ultimate public health policy recommendation.

The VCAG is a relatively new initiative so as yet there are very few biological control approaches under evaluation, but moving forward it will be important for novel biological control interventions to be submitted to the VCAG at an early stage of development to guide the R&D. The current framework for evaluation requires a substantial body of evidence, including at least some indication of the epidemiological outcome. Approval depends on whether the intervention is considered “efficacious for some defined public health purpose (in disease prevention through vector control) and in some defined circumstances” and whether it will be “useful to and feasible for its intended users”. One of the challenges with these criteria is the evaluation technologies that might not perform sufficiently well in terms of efficacy or cost to justify stand-alone use, but might nonetheless make a significant contribution in the context of broader resistance management or IVM strategies. For example, a parasite or pathogen might not be sufficiently cost-effective to use as a direct alternative for a chemical product, but its value might come from use in a rotation strategy where it slows evolution of insecticide resistance and prolongs the useful lifespan of the cheaper chemical. This situation calls for the need for ‘value-based’, rather than simple ‘cost-based’ decision-making.

Finally, in most settings, ultimate adoption of an intervention requires regulatory approval at the national level. Most countries have established regulatory frameworks for evaluating pesticides but equivalent frameworks for biological control agents are often lacking or inconsistent. Additional challenges can occur when a particular technology does not fit exactly within any existing regulatory framework, as was the case for initial introductions of *Wolbachia* trans-infected *Aedes agypti* into Australia (Murray et al. 2016). Complexities will likely increase much further if the approach involves genetic modification. Important to recognize here is that extra layers of risk assessment and potentially uncharted regulatory pathways could add years to the implementation timeline. This concern does not mean that transgenic or paratransgenic approaches are not worth pursuing, but it creates additional challenges for whether such technologies might ultimately be able to make a contribution to control, and increases the burden on the initial justification for development.

## Conclusions

There are many papers published every year reporting some new idea that “could play a role in future control of malaria vectors”, including (but definitely not restricted to) prospective biological control tools. Yet it is difficult to think of anything genuinely novel that has made it into wide scale operational use in the last 20 years. There are multiple reasons for this limited impact. To take a product from initial concept through to operational use usually requires years of R&D. There are very few opportunities for large grants, and maintaining continuous support over multiple small grants is difficult, particularly when there is a large stochastic element to funding. Sustaining the research effort through this process is also challenging, especially in academic environments where promotion and tenure processes tend to orient researchers towards short-term outputs such as publications and patents, rather than longer-term outcomes such as products or changes in practice. Yet potential industry partners want technologies to be developed as far as possible so that they know what they’re getting and there is little risk. Product development partnerships such as the Innovative Vector Control Consortium aim to help bridge this translational divide and their portfolio is now extending beyond conventional chemical approaches to include novel paradigms. More broadly, new initiatives, such as Innovation to Impact (http://innovationtoimpact.org/), are being developed to address challenges across all segments of the vector control pathway including innovation, evaluation (assessment for safety, efficacy and quality), registration, procurement, and impact. However, these initiatives are still very product/market-driven and there are additional challenges for knowledge-driven approaches that fall outside conventional public health paradigms. It is not clear, for example, how a strategy based on augmentation of natural enemies, or manipulation of a house or environmental feature fit within this product-oriented landscape. To some extent there are parallels here with the ‘top down’ implementation pathways that have promoted wide scale use of chemical pesticides in conventional agriculture, versus more participatory ‘bottom-up’ approaches, which can enable development of more diverse, knowledge-intensive IPM strategies tailored to the local context (Thomas et al. 2012).

Given these challenges, there is a need for realism and some critical self-evaluation of how and when a new tool might contribute to vector control, an argument not limited to biological control. If there is a genuine expectation to move from innovation to implementation, researchers and funding agencies should be asking whether the technology or approach is likely to have an epidemiological impact, is there a need for it or is it simply technology-driven, can it be made operational in the field, what will it take to be implemented within existing control frameworks (which includes considerations of logistics, capacity, cost and value), what are the regulatory hurdles, how much money will it cost to develop and how long will it take? This might seem like a demanding list and it is not the intention to put barriers in front of fundamental research or to constrain technology innovation. However, the lack of any genuinely novel control tool on the ground in that last 20 years points to a need for critical assessment of prospective approaches and the mechanisms of research translation. On the positive side, biological control has established itself in agriculture and environmental management in spite of very similar challenges of limited funding, demanding timelines, regulatory hurdles, operational constraints, complex cost-benefit relationships (Cock et al. 2010), etc. Can some of this knowledge and experience be transferred to public health?
